# Advancing Pediatric Dose Scaling: Strategies, Modeling Approaches, and Clinical Applications

**DOI:** 10.3390/ph19071090

**Published:** 2026-07-15

**Authors:** Rachel A. Kudgus Lokken, Sílvia M. Illamola, Kathleen M. Job, Hesham S. Al-Sallami, Geert W. ‘t Jong, David M. Reith, Angela K. Birnbaum, Catherine M. Sherwin

**Affiliations:** 1 Allucent, Cary, NC 27513, USA; rachel.kudgus@allucent.com; 2 Department of Experimental and Clinical Pharmacology, College of Pharmacy, University of Minnesota, Minneapolis, MN 55455, USA; sillamol@umn.edu (S.M.I.); birnb002@umn.edu (A.K.B.); 3 Division of Clinical Pharmacology, Department of Pediatrics, University of Utah School of Medicine, Salt Lake City, UT 84108, USA; kate.job@hsc.utah.edu; 4 College of Pharmacy, QU Health, Qatar University, Doha P.O. Box 2713, Qatar; h.al-sallami@qu.edu.qa; 5 Children’s Hospital Research Institute of Manitoba, Rady Faculty of Health Sciences, University of Manitoba, Winnipeg, MB R3E 3P4, Canada; geert.tjong@umanitoba.ca; 6 Faculty of Medicine, University of Otago, Dunedin 9054, New Zealand; david.reith@otago.ac.nz; 7 Center for Clinical and Cognitive Neuropharmacology, College of Pharmacy, University of Minnesota, Minneapolis, MN 55455, USA; 8 Department of Medical Laboratory Sciences, College of Pharmacy, University of Minnesota, Minneapolis, MN 55455, USA; 9 Internal Medicine, UWA Medical School, The University of Western Australia, Perth, WA 6009, Australia; 10 Differentia Biotech Ltd., South San Francisco, CA 94080, USA; 11 Department of Pharmacology and Toxicology, Wright State University Boonshoft School of Medicine, Dayton, OH 45435, USA

**Keywords:** pediatric pharmacokinetics, dose scaling, allometric scaling, ontogeny, PBPK modeling, population pharmacokinetics, model-informed drug development (MIDD), neonatal, pharmacometrics

## Abstract

**Background/Objectives**: Selecting appropriate doses for pediatric patients remains one of the most complex challenges in drug development because developmental changes in physiology, metabolism, organ function, and pharmacodynamics substantially influence drug exposure and response. This review summarizes current evidence-based approaches to pediatric dose selection across the developmental continuum and evaluates contemporary model-informed strategies for individualized dosing. **Methods**: A narrative review of the literature was conducted focusing on pediatric dose-scaling methodologies, developmental pharmacology, physiologically based pharmacokinetic (PBPK) modeling, population pharmacokinetic (PopPK) approaches, exposure–response analysis, therapeutic drug monitoring, and regulatory extrapolation frameworks. Special populations and clinical scenarios relevant to pediatric dose optimization were also evaluated. **Results**: Simple body weight-based scaling from adult doses inadequately accounts for developmental changes in drug disposition and response. Allometric scaling combined with maturation functions provides improved dose prediction in neonates and infants, while PBPK and PopPK modeling support mechanistic and data-driven dose optimization across pediatric age groups. Fat-free-mass (FFM)-based scaling is preferred over total body weight for many drugs in children with obesity. Additional considerations including obesity, biologics, formulation and excipient safety, pharmacogenomics, critical illness, therapeutic hypothermia, extracorporeal support, therapeutic drug monitoring, and drug–drug interactions substantially influence pediatric dosing strategies. Regulatory frameworks including ICH E11A increasingly support model-informed pediatric extrapolation and precision dosing approaches. **Conclusions**: Pediatric dose selection has evolved from empirical weight-based dosing toward integrated model-informed strategies incorporating developmental physiology, pharmacometrics, and regulatory science. Allometry, maturation functions, FFM-based scaling, PBPK, PopPK, and therapeutic drug monitoring provide complementary tools for rational pediatric dose optimization, although drug- and pathway-specific validation remains essential, particularly in neonates and critically ill children.

## 1. Introduction

Pediatric dose scaling refers to the set of methods used to translate dosing information from adults or older children to younger pediatric populations, accounting for differences in body size, organ maturation, and drug disposition [1,2]. Dose selection is one of the most challenging aspects of pediatric drug development, with clinical pharmacology playing a central role [3]. The widespread practice of calculating pediatric doses by simple body weight-based scaling from adult doses fails to account for the ontogeny of drug-metabolizing enzymes, transporters, and maturation of organ function in neonates and infants [4,5]. Different metabolic pathways and organ systems also mature at different rates. Without thorough knowledge of drug disposition in these populations, clearance (CL) and dose predictions may be inaccurate.

Ideally, pharmacokinetic (PK) and dose-ranging studies would provide direct evidence for dose–exposure relationships across pediatric age groups [4]. However, the sample sizes required for complete characterization are often infeasible due to ethical restrictions, limited blood volume, and the vulnerability of pediatric populations.

This review discusses common dose-scaling strategies for pediatric patients, examines the physiological basis for developmental changes in drug handling, and evaluates model-informed approaches, including physiologically based pharmacokinetic (PBPK) modeling and population pharmacokinetics (PopPK). A workflow for applying these methods across drug development phases is presented. The focus is on dose selection during drug development and regulatory labeling rather than bedside administration or medication-error processes, which involve distinct clinical and systems-level considerations.

While each of the methods discussed here has been reviewed individually [6,7,8], this article integrates them into a single comparative framework spanning the pediatric age continuum, aligns that framework with the 2024–2025 ICH E11A and FDA/EMA regulatory updates [9,10,11], and translates the synthesis into practical, age- and clearance-pathway-specific decision rules intended for use during dose selection and labeling. This narrative review was informed by the literature identified through PubMed/MEDLINE and Embase searches conducted up to April 2026, using combinations of terms including ‘pediatric pharmacokinetics,’ ‘dose scaling,’ ‘allometric scaling,’ ‘maturation function,’ ‘ontogeny,’ ‘PBPK,’ ‘population pharmacokinetics,’ ‘exposure-response,’ and ‘pediatric extrapolation.’ Additional sources were identified from the reference lists of key articles and from regulatory guidance documents. Articles were selected on the basis of relevance to pediatric dose scaling, regulatory significance, and recency. As a narrative rather than systematic review, literature selection reflects the authors’ domain expertise and is not based on a pre-specified search protocol; this is acknowledged as a limitation in Section 7.

For consistency, three qualifying terms are used throughout this review with deliberately distinct meanings. “Recommended” is reserved for guidance that is endorsed by a regulatory authority or formal guideline (for example, an approach specified in ICH, FDA, or EMA documents). “Preferred” denotes the approach the authors judge most suitable for a given age group or clearance pathway on the weight of published evidence and regulatory experience, without implying a formally graded ranking. “Optimal” is used only in the technical, method-specific sense (for example, an optimal sampling design or an optimal estimator) and is not used as a general synonym for “preferred.” The noun “recommendation(s),” including the title of Section 8 and references to the practical advice offered in this review, is used in its ordinary sense to denote the authors’ guidance and is distinct from the technical adjective “recommended” defined above. Where the supporting evidence is limited or consensus has not been reached, this is stated explicitly rather than conveyed through these terms, and the practical recommendations in Section 8 carry evidence-strength qualifiers on this basis.

## 2. Physiological Basis for Pediatric Dose Scaling

This section summarizes the developmental physiology underpinning pediatric dose scaling. Rather than presenting new data, it is organized to link each ontogenic process directly to the scaling method and age group for which it is most decision-relevant, providing the mechanistic basis for the comparative recommendations in Section 3 and Section 5.

The underlying physiology is well described in the literature and is not claimed to be novel; the contribution here is the explicit pairing of each developmental process with the scaling decision it constrains. Specifically, drug-metabolizing enzyme ontogeny is mapped to the maturation functions required for hepatically cleared drugs in the first one to two years of life; plasma protein-binding ontogeny is mapped to the handling of highly bound compounds and to unbound-fraction shifts in early infancy; and absorption ontogeny is mapped to oral dosing decisions in neonates and young infants. Presenting the physiology in this decision-oriented form, rather than as a standalone developmental-pharmacology summary, is what differentiates this review from existing reviews and is intended to make the basis for the method recommendations in Section 3 and Section 5 transparent.

Children have historically been described as “therapeutic orphans,” reflecting inadequate attention to pediatric-specific drug development [12]. A fundamental principle in pediatric pharmacology is that children are not simply small adults; neonates, in particular, are physiologically distinct, with still-maturing drug-disposition pathways [1]. The problems inherent in scaling adult drug doses to children have been well documented, with simple proportional adjustments frequently resulting in therapeutic failure or toxicity [2].

Developmental pharmacology encompasses age-related changes in absorption, distribution, metabolism, and excretion (ADME) that collectively determine drug exposure in pediatric patients [13,14]. These ontogenetic changes are particularly pronounced during the first two years of life, when rapid maturation of organ systems occurs alongside substantial changes in body composition [15]. Understanding the physiological basis for these differences is essential for rational dose selection across the pediatric age continuum.

Drug-metabolizing enzyme ontogeny is an important source of PK variability in children [16]. The developmental expression patterns of multiple cytochrome P450 enzymes (CYPs), UDP-glucuronosyltransferases (UGTs), and other metabolic pathways vary substantially by isoform. CYP3A7 predominates in fetal and neonatal life, then transitions to CYP3A4, with activity that increases substantially during infancy and generally approaches near-adult levels within the first 1–2 years of life, although maturation trajectories vary by substrate and methodology [17,18]. CYP2D6 follows a more variable trajectory, with adult-level activity typically reached in early childhood, though timing varies substantially by genotype. Individual UGT isoforms can also follow distinct developmental trajectories. These enzyme-specific maturation patterns preclude the application of a single correction factor across all hepatically cleared drugs. While renal maturation is often more predictable and quantifiable, hepatic clearance pathways exhibit greater inter-drug and inter-individual variability, limiting the generalizability of maturation functions.

Plasma protein binding ontogeny is a further determinant of pediatric drug exposure that is independent of CL maturation. Albumin concentrations at birth approximate 75 to 80% of adult values, while alpha-1-acid glycoprotein (AAG) concentrations are roughly half of adult levels in term neonates and rise gradually over the first year of life [19]. For drugs that are extensively bound to AAG, this developmental difference can elevate the unbound fraction in early infancy and alter both the volume of distribution (Vd) and the CL of restrictively cleared compounds (i.e., those with low hepatic extraction ratio). AAG is also an acute-phase reactant, so concomitant infection or inflammation introduces additional within-patient variability that is particularly relevant in critically ill infants [20]. In neonates, endogenous bilirubin and free fatty acids can additionally compete for albumin binding sites, increasing the free fraction of highly albumin-bound drugs and contributing to the historical concern about kernicterus risk from displacing agents. Quantitative models of binding-protein ontogeny have been incorporated into pediatric PBPK platforms, and accounting for these developmental shifts in unbound fraction is essential for accurate prediction of exposure for highly bound compounds, particularly in the first year of life.

Oral absorption and bioavailability also follow distinct developmental trajectories that are clinically relevant for orally administered drugs. Gastric pH is near-neutral at birth, decreases over the first weeks of life, and reaches adult acid secretion patterns by approximately two to three years of age, with corresponding effects on the dissolution and stability of acid- and base-labile compounds [13]. Intestinal motor activity, particularly proximal duodenal motility, matures postnatally and is more variable in preterm and term neonates than in older children, whereas mean gastric emptying time does not show a consistent age effect [21,22]. Bile acid pool size and biliary function mature postnatally, affecting the solubilization and absorption of lipophilic drugs and lipid-based formulations [21]. Intestinal drug-metabolizing enzymes (notably CYP3A4) and efflux transporters (P-glycoprotein, breast cancer resistance protein (BCRP)) show age-dependent expression patterns that influence the oral bioavailability of substrate drugs [23]. Collectively, these developmental changes can produce age-dependent differences in oral bioavailability that are not captured by simple body-size scaling and warrant explicit consideration when selecting pediatric oral doses, particularly in the first year of life.

## 3. Dose Scaling Methods

The scaling methods described in this section are individually well established. Their treatment here is deliberately comparative: each is presented with its mechanistic rationale, advantages, and limitations so that the relative suitability of each method across age groups (Section 5.1) and the practical decision rules (Section 8) follow directly from a single, consistent foundation. The intended contribution of this review is therefore not the description of any individual method, each of which has been reviewed elsewhere, but the explicit mapping of methods to one another across the full pediatric age continuum: for each approach, we specify the age range and clearance pathway over which it is most defensible, the conditions under which it should be replaced or supplemented by another method, and how the methods combine in sequence during development. This comparative, decision-oriented synthesis, carried through to the age-by-method matrix and the practical rules in Section 8, is the element that distinguishes the present treatment from the predominantly method-by-method descriptions available in the existing literature.

### 3.1. Size Descriptors

Multiple size descriptors have been proposed for pediatric dose scaling, each with distinct advantages and limitations (Table 1). Total body weight (TBW) is the most commonly used metric due to its simplicity. However, linear weight-based scaling assumes a proportional relationship between body size and drug CL that does not hold across the full pediatric age range [2].

**Figure 1:**
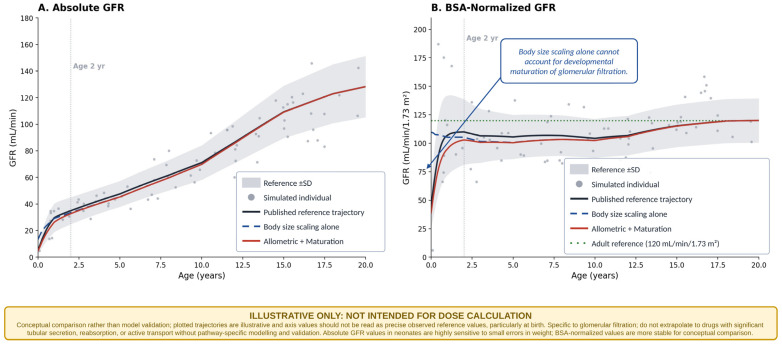
Glomerular filtration rate (GFR) ontogeny from birth to 20 years of age in term-born individuals. (**A**) Absolute GFR (mL/min) increases monotonically with somatic growth. (**B**) BSA-normalized GFR (mL/min/1.73 m^2^) is low at birth, rises rapidly through infancy, and approaches the adult reference (≈120 mL/min/1.73 m^2^) by about 2 years of age. In both panels, two model predictions are compared with an observed mean reference trajectory (black) interpolated from published pediatric reference values [27,41,42]. The blue dashed curve is allometric scaling alone (GFR = GFR_adult_ × (WT/70)^0.75^); the red solid curve adds a Rhodin-type sigmoidal maturation function (F_maturation_ = PMA^Hill^/(TM_50_^Hill^ + PMA^Hill^); TM_50_ ≈ 47.7 weeks postmenstrual age, Hill ≈ 3.40) [27]. The two predictions converge once maturation approaches adult values (vertical dashed line, ≈2 years) but diverge sharply in early life: under the illustrative assumptions used here, the illustrative body-size-only model substantially overpredicts neonatal filtration capacity, giving a BSA-normalised value of about 105 to 110 against measured values of approximately 20 mL/min/1.73 m^2^ at birth, rising to roughly 40 by the end of the first week, whereas the maturation-corrected curve tracks the observed rise. The grey band is ±1 SD and broadens in early life to reflect the higher inter-individual variability typically observed in neonates and infants; grey points are illustrative synthetic samples representing simulated variability around the reference trajectories, not observed patient-level data. This is a conceptual comparison rather than a model validation: GFR is used as a marker of filtration capacity, not as an estimate of drug clearance, and the figure is specific to glomerular filtration. It is not intended for dose calculation and should not be extrapolated, without pathway-specific modeling and validation, to drugs undergoing significant tubular secretion, reabsorption, or active transport. Absolute GFR values in neonates are highly sensitive to small errors in weight; BSA-normalized values are more stable for conceptual comparison. Abbreviations: BSA, body surface area; WT, body weight; PMA, postmenstrual age; TM_50_, age at which maturation reaches 50% of adult value; F_maturation_, maturation factor; SD, standard deviation.

To illustrate how these methods map into practice, a worked example of each approach is given below:

- Simple mg/kg dosing: paracetamol (acetaminophen) in a 25 kg, 8-year-old child with normal body composition, dosed at 15 mg/kg per dose, where the broad therapeutic index and approximately linear PK–weight relationship in this age range make linear weight-based dosing acceptable.
- BSA-based dosing: a conventional cytotoxic such as methotrexate dosed per m^2^ in pediatric oncology, a historical practice retained largely for continuity with established protocols rather than because BSA outperforms allometry.
- Allometric (0.75) scaling: predicting the clearance of a renally cleared drug in a 4-year-old from a 70 kg adult reference using CL = CLstd × (WT/70)^0.75^, which is generally adequate beyond about 2 years of age without an additional maturation term.
- Allometric scaling plus a maturation function: gentamicin or another renally cleared aminoglycoside in a term neonate, where a size term is combined with a postmenstrual-age-driven sigmoidal maturation function (for example a Rhodin-type GFR maturation function, Figure 1) to capture renal maturation that allometry alone would miss [27].
- FFM-based scaling: propofol infusion-rate selection in an obese adolescent, where scaling to fat-free mass estimated with the Al-Sallami equation tracks clearance more closely than total body weight and avoids the over-dosing that TBW-based scaling can produce [28].
- PBPK modeling: first-in-pediatric dose projection for a CYP3A4 substrate, building an age-specific mechanistic model that is first verified against adult data and then extrapolated stepwise into successively younger pediatric strata.
- PopPK modeling: vancomycin dose individualization from sparse therapeutic-drug-monitoring samples in critically ill children, using a covariate model in which clearance is scaled allometrically and matured by postmenstrual age to support Bayesian dose adjustment [31].

Body surface area (BSA) has been used historically, particularly for chemotherapeutic agents, based on the assumption that BSA correlates with metabolic rate and organ function [6,32]. However, BSA-based scaling in children is historically derived rather than mechanistically grounded, and the BSA estimation equations themselves were developed in adult populations with little or no pediatric validation. BSA-based dosing offers no clear advantage over allometric approaches, introduces avoidable calculation complexity, and is increasingly considered less favored for new pediatric applications. Allometric scaling combined with maturation functions is generally preferred for many small-molecule applications, although biologics and transporter-driven drugs may warrant pathway-specific approaches [33].

Body composition changes substantially throughout childhood, with the proportion of body water, fat mass, and lean tissue varying by age and developmental stage [15]. For many compounds, particularly those with distribution and elimination patterns linked to lean tissue mass, drug CL may correlate more closely with fat-free mass (FFM) than with total body weight (TBW), supporting FFM as a potentially superior size descriptor [30]. Equations for estimating FFM in children have been developed and validated, enabling application of FFM-based scaling in pediatric populations, though predictive performance remains drug- and age-specific [28]. The extension of allometric theory to incorporate normal fat mass provides a physiologically grounded framework for dose adjustment in obesity and across body compositions [34]. More recent work has extended this approach by deriving consistent equations for FFM, CLCr, and glomerular filtration rate that apply from neonates through adults, providing a unified framework that links body composition descriptors to renal function within a single modeling structure [35].

### 3.2. Allometric Scaling

Allometric scaling applies mathematical relationships derived from comparative physiology to predict PK parameters across species and body sizes. The theoretical basis for allometric scaling in PK derives from observations that metabolic rate scales with body weight to the power of approximately 0.75 across mammals [26]. This relationship has been formalized as a size standard for PK, enabling systematic comparison of CL values across individuals of different sizes [36].

For CL prediction, the standard allometric equation (Equation (1)) takes the form

CL = CL_std_ × (WT/WT_std_)^0.75^ (1)

where CL is the predicted clearance, CL_std_ is the clearance of a standard-sized reference individual, WT is the individual’s body weight, and WT_std_ is the reference body weight [36,37]. WT_std_ is conventionally fixed at 70 kg as a pragmatic standard adopted for inter-study comparability rather than a population mean or biologically derived constant; real-world adult reference weights vary by sex, population, and region [6,32].

The optimal allometric exponent remains subject to debate, with some investigators proposing age-dependent or drug-specific exponents rather than a fixed value of 0.75 [38,39]. Comparative analyses have demonstrated that the choice of exponent can substantially influence dose predictions, particularly in young children [38]. For many small-molecule drugs, fixed-exponent allometric scaling provides reasonable first-order predictions beyond approximately 2 years of age, although predictive performance remains drug dependent; for younger children, additional maturation functions are required [24,25]. The 0.75 exponent is a useful convention for cross-drug comparability; drug-specific exponents may fit better but rarely change clinical decisions. Allometry itself remains valid as a size descriptor at all ages; its failure in neonates reflects the absence of a maturation term, not a limitation of allometric theory.

### 3.3. Maturation Functions and Organ Ontogeny

Allometric scaling alone is insufficient for accurate dose prediction in neonates and young infants because it does not account for the functional immaturity of drug-eliminating organs [1]. To address this limitation, maturation functions are incorporated to describe the developmental time course of CL pathway activity.

Glomerular filtration, the filtration component of renal clearance, has been well characterized, with glomerular filtration rate (GFR) following a predictable maturation pattern from birth through early childhood [27]. Absolute GFR is approximately 2–4 mL/min at birth in term neonates (corresponding to approximately 20–40 mL/min/1.73 m^2^ when body-surface-area normalized), increases rapidly during the first weeks of life, and approaches adult values (normalized to BSA) by 1–2 years of age (Figure 1).

The maturation function for GFR can be described using a sigmoidal model (Equation (2)):

F_maturation_ = PMA^Hill^/(TM_50_^Hill^ + PMA^Hill^) (2)

where F_maturation_ is the fraction of adult function attained, PMA is postmenstrual age, TM_50_ is the PMA at which maturation reaches 50% of adult values (approximately 47.7 weeks for GFR), and Hill is the coefficient that describes the steepness of the maturation curve [27]. This is not the only defensible parameterisation: in a simultaneous analysis of gentamicin, tobramycin, and vancomycin from preterm neonates to adults, maturation of GFR-mediated clearance was better described by a bodyweight-dependent exponent, decreasing from approximately 1.4 in neonates to 1.0 in adults, than by a fixed allometric exponent with a separate maturation term [40].

This illustration reflects glomerular filtration specifically and should not be extrapolated to drugs undergoing significant tubular secretion, reabsorption, or active transport without pathway-specific modeling. Although the maturation framework summarized here is broadly applicable to healthy term-born infants, it is important to recognize that disease state, prematurity, nephron endowment, acute kidney injury, therapeutic hypothermia, and extracorporeal support can each substantially perturb the apparent renal ontogeny trajectory and the timing of approach to adult function; these factors are discussed further in Section 6.4. More recent work by O’Hanlon and colleagues offers a consistent set of equations that describe FFM, CLCr, and GFR across the lifespan from neonates to adults, harmonizing body size and renal function descriptors within a single modeling framework [35].

GFR estimation in children requires age-appropriate equations (Table 2). The Schwartz equation and its variants are the most widely used approaches and are recommended by the Kidney Disease Outcomes Quality Initiative guidelines [43,44,45]. The Schwartz equation uses HT (height) and serum creatinine (SCr), with the updated bedside form expressed as eGFR = 0.413 × HT/SCr. Although the original Schwartz equation tends to overestimate GFR by 20 to 25% when used with modern isotope dilution mass spectrometry (IDMS)-traceable creatinine assays [44], the updated bedside form remains the most widely used method for estimating GFR in young children. It is not, however, validated for children under 1 year of age [44]. U.S. Food and Drug Administration (FDA) guidance on PK in patients with impaired renal function provides additional recommendations for dosing adjustments based on renal function estimates [46].

Hepatic and renal clearance pathways in infants follow distinct developmental trajectories that must be considered when scaling doses of drugs eliminated by these routes [55]. Beyond glomerular filtration, tubular secretion and reabsorption processes also mature postnatally. Renal drug transporter ontogeny has been characterized through mRNA analysis and quantitative proteomics, revealing that organic anion and cation transporters reach adult expression levels at different developmental stages [56]. In neonates, gestational age and postnatal age represent independent determinants of organ maturation and CL and should be considered separately when developing covariate models or selecting maturation functions; PMA is widely used as a composite descriptor that integrates these contributions.

### 3.4. Exposure Matching

The most common approach to selecting doses in pediatric clinical trials is to match exposures to doses found safe and efficacious in adults [57]. This exposure-matching strategy assumes that disease progression and response to therapeutic interventions are similar between pediatric and adult patients. However, this assumption may not hold across all therapeutic areas, as developmental differences in disease pathophysiology, receptor expression, and pharmacodynamic (PD) responses can yield distinct exposure–response (E-R) relationships in children compared to adults.

When sufficient information demonstrates that equivalent efficacy can be achieved at exposures lower than those observed in adults, lower target exposures may be acceptable [57]. For rare pediatric diseases where no clinical data exist, initial doses are selected based on PK and toxicokinetic data from nonclinical studies, with appropriate consideration of interspecies differences in ADME and protein binding [58].

Developmental differences in receptor ontogeny, target expression, and disease biology may lead to divergent E-R relationships, necessitating pediatric-specific PD evaluation when biological similarity cannot be assumed [59]. Empirically, exposure matching does not guarantee demonstrated efficacy. In an FDA review of pediatric extrapolation programmes, 20 of 86 trials did not result in an indication, with efficacy insufficiently evaluated or not demonstrated in 13 of these despite exposure-based dose selection [57]. Pediatric inflammatory bowel disease similarly illustrates extrapolation proceeding under similarity assumptions that carried residual uncertainty about E-R equivalence [60]. These considerations are particularly important in neonates and young infants, where divergent developmental physiology, pharmacogenetic (PGx) factors, and still-maturing PD systems mandate population-specific trial design rather than direct extrapolation from older children or adults [61].

An additional limitation specific to therapeutic proteins is that adult concentration-effect or dose–response relationships for many monoclonal antibodies and other biologics are themselves poorly characterized in the pivotal data on which pediatric extrapolation is based. Where the adult E-R is incompletely defined, strict replication of adult exposure in children is neither straightforwardly achievable nor universally required, and the decision to pursue exposure matching versus pediatric-specific E-R evaluation should be made on a drug-by-drug basis [33,62].

### 3.5. Pharmacodynamic Ontogeny

Pharmacodynamic ontogeny is conceptually distinct from PK ontogeny and refers to the developmental changes in drug target expression, receptor density and coupling, downstream signaling, and homeostatic regulation that determine the response to a given drug exposure. The clinical relevance of these changes varies by therapeutic area. Receptor ontogeny has been described for several systems, although the evidence base remains heterogeneous and many pediatric PD differences are incompletely characterized. Reasonably well-supported examples include developmental changes in GABA-A subunit composition during early infancy (with implications for benzodiazepine and anesthetic effects), age-related differences in beta-adrenergic receptor density and coupling in the neonatal myocardium, and opioid receptor expression patterns that have been proposed to contribute to altered analgesic sensitivity in early life [60,63]. Developmental changes in coagulation factor concentrations affect the PD response to anticoagulants [64]. Age-dependent differences in target expression and disease biology can produce E-R curves that differ from those in adults even when PK exposure is matched. As a consequence of developmental changes in receptor expression, target density, and disease biology, exposure matching alone is insufficient when PD similarity cannot be assumed, and pediatric-specific PD evaluation should be incorporated into the development plan whenever evidence of developmental divergence exists or cannot be ruled out [10,57].

### 3.6. Pharmacogenomic Considerations

Genetic variation in drug-metabolizing enzymes and transporters is a current rather than future consideration in pediatric dose individualization, and several pediatric drug-gene pairs are already supported by evidence-based guidelines and FDA labeling. The Clinical Pharmacogenetics Implementation Consortium (CPIC) guideline for thiopurines (azathioprine, mercaptopurine, thioguanine) recommends pre-treatment genotyping for both *TPMT* (*thiopurine S-methyltransferase*) and *NUDT15* (*nudix hydrolase 15*) in any patient initiating thiopurine therapy, with genotype-directed starting-dose reductions to mitigate severe myelosuppression; this is now standard of care in pediatric acute lymphoblastic leukemia and inflammatory bowel disease [65]. Other pediatric drug-gene pairs with established CPIC guidance include *CYP2C19* and voriconazole (where poor and intermediate metabolizers exhibit substantially elevated trough concentrations), *CYP3A5* and tacrolimus (relevant to pediatric transplant dosing), and *CYP2D6* and codeine (the basis for the FDA boxed warning against codeine use in children). Importantly, genotype effects do not replace the need for ontogeny-aware dose selection: enzyme expression must mature before genotype-attributable activity differences become apparent, so PGx-guided dosing is generally most informative beyond infancy and should be integrated with developmental considerations rather than applied in isolation.

## 4. Model-Informed Approaches

### 4.1. Physiologically Based Pharmacokinetic Modeling

PBPK modeling integrates drug-specific properties with system-specific physiological parameters to predict drug disposition in target populations [7]. PBPK models represent the body as a series of anatomically and physiologically defined compartments, enabling mechanistic prediction of ADME across different populations.

A pediatric PBPK analysis follows a structured model lifecycle, and a consensus workflow has been articulated by the International Consortium for Innovation and Quality in Pharmaceutical Development (IQ) Pediatric PBPK Working Group [66]. In the first step, system-specific parameters describing the target population are assembled, including age-dependent anatomy and physiology (organ weights and blood flows, body composition, plasma protein concentrations, and glomerular filtration rate) together with the ontogeny of drug-metabolizing enzymes and transporters; these are combined with drug-specific parameters (physicochemical properties, protein binding, intrinsic metabolic and transporter kinetics, and absorption characteristics) to construct the model. The model is then verified by confirming that it recovers observed adult pharmacokinetics and, where available, any existing pediatric data, before it is used predictively. Predictive performance is judged against pre-specified acceptance criteria with predicted exposure metrics most commonly required to fall within 1.5- to 2-fold of observed values, an accuracy generally considered acceptable for dose selection and demonstrated for well-characterized substrates such as those metabolized by CYP3A4 [67]. Extrapolation then proceeds in a stepwise manner from older to younger age groups, scaling the verified adult model through successive pediatric strata; predictive uncertainty is greatest in neonates and preterm infants, where ontogeny data are sparsest and physiological variability is largest, so predictions in these groups warrant the most conservative interpretation and, where feasible, confirmation against emerging clinical data. Important limitations constrain routine application, including the need for formal model qualification, the limited availability of neonatal and preterm ontogeny data for enzymes and transporters, the cost of commercial PBPK software, and the requirement for specialized modeling expertise.

The application of PBPK modeling in pediatric drug development has expanded substantially over the past decade, with increasing regulatory acceptance for dose selection and labeling decisions [30,68]. Knowledge-driven PBPK approaches can guide first-in-children dosing when pediatric PK data are limited or unavailable [69]. The integration of modeling and simulation in pediatric drug therapy enables the application of pharmacometric principles to define appropriate doses for children [70].

The role of modeling and simulation in Pediatric Investigation Plans (PIPs) has been formally recognized by the European Medicines Agency (EMA) [71], and the US Food and Drug Administration has published guidance on model-informed drug development (MIDD) that specifically addresses pediatric applications [72]. FDA reviews of PBPK submissions have demonstrated the utility of these approaches for pediatric dose selection across multiple therapeutic areas [68].

For preterm neonates, gestational age-specific PBPK models have been developed and applied. Abduljalil et al. [73,74] integrated developmental physiology and ontogeny data to build (Part I) and apply (Part II) a preterm-specific PBPK model that addresses reduced organ function, altered body composition, and still-maturing elimination pathways. Examples of PBPK and PopPK modeling applications in pediatric drug development are summarized in Table 3.

Pediatric PBPK applications have continued to expand rapidly. A 20-year analysis identified a 33-fold increase in pediatric PBPK publications between 2005 and 2020, driven primarily by clinical and drug development applications [84]. Recent studies continue to demonstrate acceptable predictive performance for well-characterized compounds and expanding regulatory confidence in PBPK-informed pediatric dose selection [67,85]. In parallel, population PBPK approaches have been proposed to address ontogeny uncertainty by simultaneously estimating age-dependent system parameters in rare pediatric diseases where reference physiological data remain limited [86]. Consensus reviews emphasize that PBPK is widely regarded as a leading mechanistic approach for pediatric PK prediction, while allometric and maturation-based methods continue to perform well for selected drugs and age ranges; combinations of approaches are increasingly used rather than choosing a single method [59]. For neonatal populations specifically, current advances and remaining data gaps in physiology, ontogeny, and disease-related variability have been comprehensively reviewed [87]. Pragmatic, model-informed dosing approaches that combine published compound models with virtual pediatric physiology platforms are increasingly applied to support real-world clinical care, particularly for off-label use where dedicated pediatric PK studies are unlikely to be conducted [88].

### 4.2. Population Pharmacokinetic Modeling

PopPK approaches are the most commonly used methodology in neonatal drug development studies [89]. PopPK uses nonlinear mixed-effects modeling and allows analysis of sparse and unbalanced data, an important feature for neonates, where ethical and practical constraints limit sampling. The role of population PK/PD modeling in pediatric clinical research has been extensively reviewed [8], with specific guidance available on analytical approaches and common pitfalls [24]. The PopPK examples in Table 3 illustrate these applications across therapeutic areas.

Despite identical doses, highly variable concentration-time profiles are observed across pediatric patients. Variability is a substantial factor, with systematic differences accounted for by incorporating covariates representing patient factors that co-vary with PK parameters [90]. Models often explain only a portion of between-patient variability, and unexplained variability is typically higher in neonates than in older children.

Pediatric PopPK model development proceeds in stages. A structural model describing the concentration-time data (for example, the number of disposition compartments and the absorption and elimination characteristics) and a statistical model describing between-subject and residual variability are first established, after which a covariate model is built to explain systematic sources of variability [24]. In pediatric analyses, body size and organ maturation are the dominant covariates and are most informative when entered as mechanistic rather than purely empirical functions: clearance and volume are commonly scaled allometrically to body size, and a maturation function driven by PMA is added to describe the developmental time course of eliminating-organ function, mirroring the size-and-maturation framework used for allometric scaling [6,27,32]. Expressing covariates in this physiologically grounded form improves the plausibility of predictions in age ranges that are sparsely sampled and reduces the risk of overfitting. The resulting model should be evaluated by both internal and, where feasible, external validation. Internal methods include goodness-of-fit diagnostics, visual predictive checks (VPCs) that compare simulated and observed concentration distributions, nonparametric bootstrap to assess parameter precision and stability, and normalized prediction distribution errors (NPDEs) to test the adequacy of the model and its variability structure; external validation against an independent dataset provides the strongest evidence of predictive performance. Finally, extrapolation beyond the studied population should be approached cautiously: a model developed in older children may not reliably predict exposure in neonates or preterm infants, where maturational processes, disease-state effects, and unexplained variability are greatest, and predictions outside the observed ranges of age, weight, and organ function require explicit qualification and, ideally, confirmation against emerging data.

### 4.3. Exposure–Response Modeling

E-R studies support dose selection in pediatric efficacy trials and dose recommendations for regulatory approval [91]. Doses can be optimized based on exposure-efficacy and exposure-safety characteristics, which define acceptable exposure ranges as targets for drug dosing. Understanding E-R relationships for efficacy and safety in both adults and children helps determine whether changing pediatric doses or creating new strengths is warranted. For drugs with wide therapeutic margins, modest increases in pediatric concentrations may not represent safety concerns [92].

## 5. Translational and Regulatory Applications

Building on the methodological foundations of allometric scaling, maturation functions, and model-informed approaches described in Section 3 and Section 4, this section addresses how these tools are applied translationally to support pediatric drug development decisions. The choice among available scaling approaches is age- and mechanism-dependent, and the optimal strategy varies across the drug development lifecycle. We compare the relative utility of dose-scaling approaches across the pediatric age continuum, outline a structured workflow for pediatric dose selection across drug development phases, and summarize current regulatory frameworks for pediatric extrapolation and labeling.

### 5.1. Comparison of Dose Scaling Approaches

**Figure 2:**
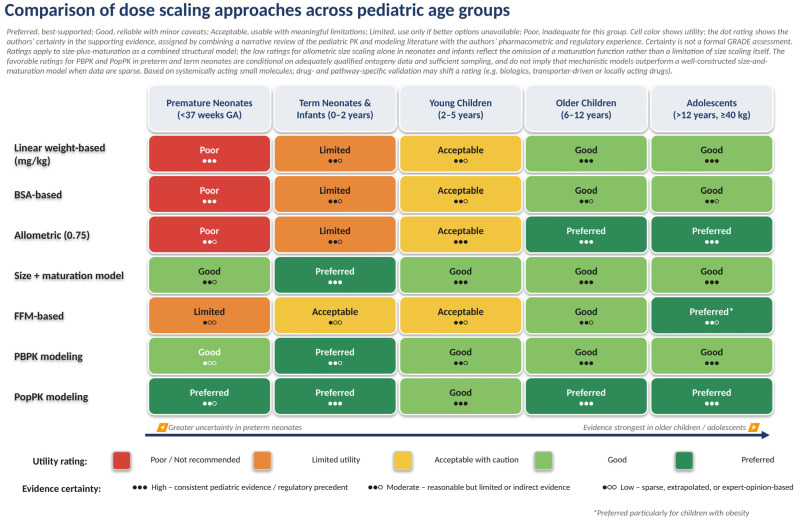
Comparison of dose scaling approaches across pediatric age groups. Cell colour indicates the utility of each approach for each age group; the dot rating indicates the authors’ certainty in the supporting evidence (three filled dots, high; two filled dots, moderate; one filled dot, low). Utility ratings are defined as follows: Preferred, best-supported; Good, reliable with minor caveats; Acceptable, usable with meaningful limitations; Limited, use only if better options are unavailable; Poor, inadequate for this group. Certainty ratings were assigned by combining a narrative review of the paediatric PK and modelling literature with the authors’ pharmacometric and regulatory experience and do not constitute a formal graded (Grading of Recommendations Assessment, Development and Evaluation; GRADE) assessment. Ratings for size plus maturation apply to the combined structural model; the low ratings for allometric size scaling alone in neonates and infants reflect the omission of a maturation function rather than a limitation of size scaling itself. The higher ratings for PBPK and PopPK in preterm and term neonates are conditional on adequately qualified ontogeny data and sufficient sampling, and do not imply that mechanistic models outperform a well-constructed size-and-maturation model when data are sparse; in practice few neonatal PBPK models are formally qualified, and many regulatory submissions continue to rely on population PK combined with maturation functions rather than PBPK alone. Ratings are based on systemically acting small molecules; drug- and pathway-specific validation may shift a given rating (for example, biologics, transporter-driven, or locally acting drugs). Abbreviations: BSA, body surface area; FFM, fat-free mass; PBPK, physiologically based pharmacokinetic; PopPK, population pharmacokinetic; GA, gestational age.

The relative utility of different dose scaling approaches varies across the pediatric age continuum (Figure 2). For premature neonates, PBPK modeling with preterm-specific physiological parameters is often the preferred mechanistic approach, provided that sufficient mechanistic data are available for model development and validation [73,74]. The preferred ratings for PBPK and PopPK in preterm neonates are conditional on adequately qualified ontogeny data and sufficient sampling; in practice, few neonatal PBPK models are formally qualified, and many regulatory submissions continue to rely on population PK combined with maturation functions rather than PBPK alone. Simple allometric scaling generally performs poorly in this population due to incomplete maturation of organ function [1]. For term neonates and infants, allometric scaling combined with maturation functions is widely used and often preferred [6,32]. For older children and adolescents, standard allometric scaling or weight-based (mg/kg) dosing is generally adequate, particularly for drugs with broad therapeutic indices [24,25].

These comparative judgements underpin the recommendations made throughout this review, so it is important to be explicit about how they were reached and how much confidence they carry. Each cell rating in Figure 2 was assigned by combining a narrative review of the published pediatric pharmacokinetic and modeling literature for each method and age group with the authors’ collective experience in pediatric pharmacometrics and regulatory practice; where the literature was sparse or conflicting, particularly for preterm and term neonates, ratings were weighted toward documented predictive performance and regulatory precedent and flagged as carrying greater uncertainty. In broad terms, the utility categories were distinguished as follows: Preferred denotes the method best supported for that age group and clearance pathway by consistent predictive performance and regulatory precedent; Good denotes reliable performance with only minor caveats; Acceptable with caution denotes usable performance offset by meaningful limitations or sparse pediatric validation; Limited denotes substantial limitations such that the method is appropriate only when better-supported options are unavailable; and Poor/Not recommended denotes predictive performance judged inadequate for that group. These categories are qualitative and were applied using the same three considerations throughout: predictive performance against observed pediatric data, the extent of supporting evidence, and regulatory acceptance.

The matrix is therefore intended as expert, evidence-informed guidance rather than a systematic or formally graded (e.g., GRADE) assessment. Confidence is lower in the youngest age groups, particularly preterm neonates, where the evidence is sparsest; the Preferred ratings for PBPK and PopPK in these groups assume adequately qualified mechanistic data and sufficient sampling. Ratings are general, and drug- and pathway-specific validation may shift an individual rating, particularly for biologics, transporter-driven drugs, and locally acting agents.

### 5.2. Workflow for Pediatric Dose Selection

**Figure 3:**
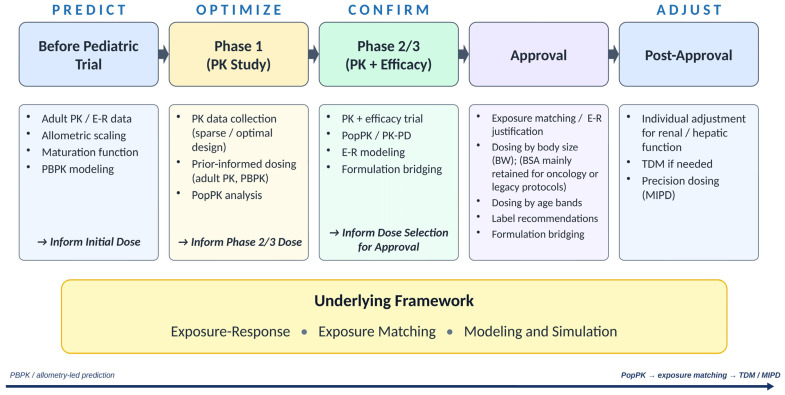
Workflow for pediatric dose selection across drug development phases. Schematic of the model-informed approach to pediatric dose selection, from initial prediction through post-approval adjustment, with modeling and simulation applied at each stage. The iterative Predict, Optimize, Confirm, and Adjust cycle moves from adult PK and exposure–response (E-R) data with allometric or PBPK predictions (Predict), through population PK characterization in early pediatric studies (Optimize) and E-R or exposure-matching analysis in efficacy trials (Confirm), to labeled dosing at approval and post-approval individualization including TDM and model-informed precision dosing (MIPD) (Adjust). Pediatric development pathways are often non-linear, with Phase 1/2 hybrid designs or stand-alone PK bridging studies common in practice [27,41,42,44,93,94]. Abbreviations: PK, pharmacokinetic; E-R, exposure–response; PBPK, physiologically based pharmacokinetic; PD, pharmacodynamic; BSA, body surface area; TDM, therapeutic drug monitoring; MIPD, model-informed precision dosing.

A structured workflow should guide pediatric dose-selection decisions across drug development phases (Figure 3). It is best understood as an iterative cycle of prediction, optimization, confirmation, and post-approval adjustment, in which modeling and simulation and exposure matching between pediatric and adult populations inform each decision, and data emerging at later stages feedback to refine earlier predictions. The key decision points are as follows:

Selection of the first-in-pediatric dose based on adult PK data: For predicting drug CL, fixed-exponent allometric scaling is generally adequate for adolescents and children older than 2 years for many small-molecule drugs, although predictive performance remains drug dependent, while maturation functions should be incorporated for pediatric patients younger than 2 years [6,32]. If no adult indication exists, doses may be selected by matching animal exposure with proper interspecies scaling [38,58]. PBPK modeling provides an additional platform for initial dose projection [7,69]. Conservative starting doses should be used when uncertainty is high.

Selection of a pediatric dose for phase 2/3 trials with pediatric and adult PK data available: The primary approach is to match adult exposure at the approved dose level [57]. Population PK modeling (as applied to amikacin and vancomycin) enables dose optimization [31]. At the same time, pharmacokinetic/pharmacodynamic (PK/PD) and E-R studies (commonly used for anti-infective and antiviral drugs) support dose selection decisions [8].

Dose selection for pediatric use: Doses should align with exposure within the target range established from adult efficacy and safety data [10]. For products approved under the Animal Rule or similar pathways, exposure matching to animals with proper scaling may be applied [58]. Formulation bridging studies may be required when pediatric-specific formulations differ from adult products [95].

Recommended dosing regimens in drug labels: Final labeling may specify dosing based on body weight (weight-band dosing), BSA, age, or a combination of age and body size (as exemplified by acetaminophen dosing guidelines) [92]. Body surface area-based labeling, although used historically for some pediatric products, is now generally discouraged for new pediatric labels in the absence of a specific mechanistic rationale, because pediatric BSA estimation equations lack adequate validation in young children and BSA does not consistently outperform allometric or weight-band approaches; weight-band or age-and-weight-band labeling is therefore preferred for most new products [6,32].

Personalized dosing for pediatric patients after approval: Individual dose adjustments based on renal or hepatic function, therapeutic drug monitoring (TDM), or other patient-specific factors may be recommended for drugs with narrow therapeutic indices or substantial PK variability [31].

### 5.3. Regulatory Applications

In pediatric drug development, modeling and simulation have been used to leverage prior information and to guide dosing strategies in subsequent clinical trials [80]. Observed clinical PK data typically supports drug approval and labeling. Still, well-validated modeling and simulation can serve as viable alternatives in certain circumstances, such as rare diseases, where dedicated PK studies are infeasible [72]. Notable regulatory approvals supported primarily by modeling include adalimumab for adolescent hidradenitis suppurativa (where no pediatric clinical trial was conducted due to disease rarity), esomeprazole dosing in pediatric gastroesophageal reflux disease (GERD), and canakinumab in children younger than two years with periodic fever syndromes [80,81,82].

Recent regulatory frameworks have formalized the role of pediatric extrapolation in drug development [10,11]. Pediatric drug development is structured around standardized age classifications defined by the International Council for Harmonisation of Technical Requirements for Pharmaceuticals for Human Use (ICH) E11/E11(R1), with closely related but not identical operational categories adopted by the FDA and EMA (Table 4) [10,96,97]. Although these age definitions are each well established, they are rarely presented together, and their points of divergence are easily overlooked when each framework is consulted in isolation. Table 4 therefore sets the three side by side to make these differences explicit. The most consequential is the upper age bound for adolescents: the FDA caps the pediatric population at younger than 17 years under 21 CFR 201.57, whereas ICH and the EMA extend adolescence to 18 years [98,99]. The EU framework further subdivides childhood into preschool and school-age strata. This harmonised comparison is particularly relevant when planning multi-region pediatric programs, where a single age definition cannot be assumed across jurisdictions.

The ICH E11A guideline on pediatric extrapolation, which reached Step 4 of the ICH process on 21 August 2024 (with the FDA final guidance issued in December 2024 and the EMA Step 5 version legally effective on 25 January 2025), distinguishes between full and partial extrapolation of efficacy from adults to pediatric populations based on the similarity of disease progression, E-R, and PD response [10]. Under this framework, exposure matching alone may be sufficient when assumptions of disease and response similarity are well supported, whereas additional pediatric-specific PD, efficacy, or safety data are required when uncertainty is greater [10]. The EMA reflection paper on extrapolation in pediatric medicine development provides complementary guidance on integrating extrapolation concepts and plans into pediatric investigation plans [11]. Updated FDA guidance on general clinical pharmacology considerations for pediatric and neonatal studies further reinforces the use of model-informed approaches, including PBPK and PopPK, as central tools for pediatric dose selection and labeling decisions [3].

## 6. Special Considerations

### 6.1. Obesity in Pediatric Dosing

In the United States, obesity affects approximately 20% of children and adolescents, presenting unique dosing challenges [102]. Volume of distribution and CL often do not increase linearly with body weight in obese individuals. Children with substantial adiposity have greater adipose tissue volume, resulting in larger Vd and prolonged elimination half-lives for lipophilic drugs that partition into adipose tissue. For example, weight-based dosing using TBW can lead to over-dosing for several drug classes in obese children. Propofol maintenance infusion rates per kilogram are lower in obese children, and weight-normalized CL or Vd differs from normal-weight controls for several other drugs reviewed in this population, including selected antibiotics (gentamicin, vancomycin) and antineoplastics (busulfan) [103]. When prescribing to overweight children, clinicians should differentiate between obesity and increased muscle mass, as body mass index (BMI) cannot distinguish between these body composition patterns. FFM-based scaling has emerged as a more appropriate approach than TBW for many drugs, although the predictive advantage of FFM is drug-, pathway-, and age-dependent and is influenced by lipophilicity, plasma protein binding, target-tissue partitioning, and transporter involvement; drug-specific evaluation remains appropriate before substituting FFM-based for TBW-based scaling; the Al-Sallami equation provides a pediatric-validated FFM estimator that has been incorporated into both empirical allometric scaling and PBPK frameworks [28]. Pharmacokinetic studies in children with obesity have shown that weight-normalized CL values may differ from those in normal-weight children for several drug classes, supporting the need for body-composition-informed dose individualization in this population [103].

### 6.2. Formulation Considerations

Developing age-appropriate pediatric dosage forms is critical for drug development success [95]. Regulatory guidance emphasizes that pediatric study plans should include formulation development across all age groups, with consideration of palatability, dose flexibility, and excipient safety. Formulation and excipient considerations act as direct constraints on the implementation of a scaled dose: the range of pediatric clearance values across age groups drives the need for multiple strengths and weight-band dose tables (Section 8), and because excipient exposure scales with the administered dose and volume, a dose that is appropriate on a pharmacokinetic basis may still be unsafe in neonates owing to cumulative benzyl alcohol, propylene glycol, or ethanol exposure [95,104,105,106]. To accommodate the wide range of dose requirements from preterm neonates to adolescents, developers may need multiple dosage strengths, age- or weight-appropriate formulations, or flexible dosing presentations that deliver the intended dose accurately while minimizing dosing errors [95,107].

Excipient safety warrants particular attention in neonates and young infants because several commonly used pharmaceutical excipients have demonstrated age-specific toxicities at exposures that are well tolerated in adults. Benzyl alcohol, used as a bacteriostatic preservative in parenteral products, has been associated with neonatal gasping syndrome and fatal metabolic acidosis in preterm and term infants exposed to cumulative doses that exceed their capacity for benzoic acid metabolism [108]. Propylene glycol, used as a co-solvent in intravenous formulations of phenobarbital, phenytoin, lorazepam, and several antifungals, can accumulate in neonates owing to still-maturing alcohol dehydrogenase activity and renal CL, with reported toxicities including hyperosmolality, metabolic acidosis, and central nervous system depression [105]. Ethanol exposure from oral and parenteral products contributes to systemic alcohol burden in neonates and infants and may potentiate the effects of co-administered central nervous system depressants [106]. Other excipients of pediatric concern include polysorbate 80, parabens, and certain coloring agents. International pediatric formulary frameworks and the STEP (Safety and Toxicity of Excipients for Paediatrics) database now provide age-specific exposure thresholds that should be considered during formulation selection and labeling [109].

Historical pediatric dose-scaling failures illustrate the clinical consequences of applying adult-derived assumptions to infants without accounting for developmental pharmacology. Chloramphenicol gray baby syndrome in the late 1950s was caused by accumulation of unconjugated chloramphenicol in neonates owing to still-maturing UGT2B7 activity and limited renal excretion, producing cardiovascular collapse at doses adjusted only for body weight [110]. Sulfonamide-induced kernicterus reflected displacement of bilirubin from albumin by competing high-affinity binding of sulfonamides, with subsequent deposition of unconjugated bilirubin in the central nervous system of vulnerable neonates [111]. Codeine-related deaths in children who were *CYP2D6* ultra-rapid metabolizers, particularly after adenotonsillectomy, led to the FDA boxed warning against codeine use in children and remain a contemporary example of how developmental and PGx factors can converge to produce unanticipated toxicity [112]. These episodes underpin the modern emphasis on developmentally informed dose selection and excipient safety assessment.

### 6.3. Biologics and Therapeutic Proteins

Therapeutic proteins, including monoclonal antibodies (mAbs), exhibit PK properties that differ substantially from those of small molecules. Their disposition is governed by target-mediated drug disposition, neonatal Fc receptor (FcRn)-mediated recycling, and catabolism via the reticuloendothelial system rather than by hepatic CYP-mediated metabolism or renal filtration [62]. Standard allometric scaling with a fixed exponent of 0.75 may not accurately predict pediatric CL for these molecules, and empirical analyses have reported exponents of approximately 0.75 for CL and 0.8 for Vd, with greater nonlinearity observed in newborns and infants under 1 year [33]. In very young infants, additional factors such as higher extracellular water content, larger capillary surface area per tissue volume, and incomplete maturation of FcRn expression may further affect distribution and elimination, although quantitative human ontogeny data remain limited. PBPK platforms incorporating these processes have been developed for pediatric mAb dose prediction and offer a complementary approach when allometric assumptions cannot be relied upon [7,33]. A recent FDA review of biologic drug approvals from 2002 through 2021 demonstrates that PopPK analyses have been applied across a range of pediatric biologic programs to inform dosing and support extrapolation from adult or other pediatric age groups, providing concrete regulatory precedent for these approaches [113].

An additional pediatric consideration for biologics is the development of anti-drug antibodies (ADAs). The pediatric immune system itself is developmentally distinct, with age-dependent changes in innate and adaptive responses, regulatory T-cell function, and the cytokine milieu. These differences can influence ADA incidence, titer, and persistence, with potential consequences for CL, exposure, efficacy, and hypersensitivity risk [62,114]. Reported immunogenicity rates in pediatric clinical programs are not consistently comparable to adult rates for the same product, and age-stratified analyses are increasingly expected in pediatric biologic development [80]. Considerations include assay sensitivity calibrated to pediatric matrices, sampling schedules that capture early seroconversion, and the integration of ADA status into pediatric PopPK models when ADA-related variability in CL is a plausible driver of exposure differences.

### 6.4. Critical Illness and Disease-State Considerations

PK alterations in critically ill children frequently dominate over the developmental factors discussed in earlier sections. As a result, standard maturation- or weight-based dose scaling can yield clinically meaningful exposure errors in the pediatric intensive care setting. Augmented renal clearance (ARC), defined as a measured GFR exceeding age-expected values (in adults, commonly defined as a CLCr exceeding 130 mL/min/1.73 m^2^), has been reported in a substantial proportion of critically ill pediatric patients, reaching up to approximately two-thirds in selected intensive care cohorts [115], although prevalence varies markedly with case-mix, age, and the method used to measure or estimate GFR. ARC is poorly identified by standard creatinine-based estimating equations and is associated with subtherapeutic exposures of renally cleared antimicrobials, including beta-lactams, vancomycin, and aminoglycosides [116]. Conversely, neonates with hypoxic–ischemic encephalopathy treated with therapeutic hypothermia demonstrate decreased CL of multiple agents. Reported reductions are approximately 25% to 35% for gentamicin, up to 40% for amikacin, and 21% to 47% for morphine. Standard normothermic dose intervals can therefore produce sustained supratherapeutic exposures [117]. Extracorporeal membrane oxygenation (ECMO) introduces a further set of disposition perturbations: increased circuit volume expands the apparent Vd, and circuit components sequester lipophilic and highly protein-bound drugs (notably midazolam, fentanyl, and several antifungals), producing pronounced and time-varying alterations in both CL and Vd that are difficult to capture with conventional pediatric dose scaling [118]. These disease-state factors, together with sepsis-related changes in protein binding, tissue perfusion, and hepatic blood flow, argue strongly for TDM and model-informed precision dosing (MIPD) rather than empirical weight-based dosing in critically ill pediatric populations.

### 6.5. Therapeutic Drug Monitoring

Therapeutic drug monitoring plays a central role in pediatric dose optimization, particularly for narrow-therapeutic-index drugs and in populations where PK variability is high. In pediatric practice, TDM is most established for the aminoglycosides and vancomycin, where target attainment based on trough concentrations or area under the curve has been linked to both efficacy and avoidance of nephrotoxicity and ototoxicity [31]. Evidence-based aminoglycoside dosing strategies incorporating therapeutic drug monitoring and PK/PD principles have also been developed for pediatric cystic fibrosis populations, further illustrating the importance of individualized dosing approaches in children [52]. Bayesian TDM platforms now allow individualized dose adjustment after a single concentration in critically ill neonates and children, addressing the limitations of conventional weight-based regimens in the setting of ARC, hypothermia, or extracorporeal support [31]. TDM is also routine for anticonvulsants (including phenobarbital, phenytoin, carbamazepine, lamotrigine, and levetiracetam in selected settings), for several immunosuppressants in pediatric transplantation (tacrolimus, cyclosporine, mycophenolic acid, sirolimus), and for selected pediatric oncology agents (methotrexate, busulfan) [119,120]. The convergence of pediatric TDM with PopPK models and electronic health record (EHR) integration is enabling MIPD in routine clinical care [121]. Routine implementation nonetheless remains uneven, constrained by assay availability and turnaround time, the maturity of EHR integration, and variable adoption of Bayesian dosing platforms across centers.

### 6.6. Drug–Drug Interaction Considerations

Drug–drug interactions (DDIs) in pediatric patients can differ qualitatively and quantitatively from those in adults because the enzymes and transporters mediating the interaction are themselves developmentally regulated. The magnitude of CYP-mediated DDIs may be attenuated when the implicated enzyme has not yet reached adult activity, and may be exaggerated when a single residual pathway carries a disproportionate share of CL [122,123]. Similar developmental considerations apply to transporter-mediated interactions for substrates of P-glycoprotein, organic anion transporting polypeptide (OATP), and renal organic anion and cation transporters. Pediatric PBPK modeling has emerged as the leading tool for predicting the direction and magnitude of pediatric DDIs in the absence of dedicated clinical interaction studies, and pediatric PBPK-based DDI predictions are increasingly accepted by regulators for labeling decisions [68,85]. In neonates and infants, the combination of polypharmacy in intensive care, narrow therapeutic indices, and still-maturing elimination pathways means that the absolute risk of clinically meaningful DDIs may be higher than in older children even when relative interaction magnitudes are smaller. Anticipation of DDI risk during the development of pediatric formulations and dosing regimens is therefore an integral part of pediatric dose selection [122].

## 7. Challenges and Limitations

Despite accumulated knowledge and modeling tools for improved dosing strategies, collaborative research is warranted to address challenging issues in pediatric dose selection. A careful balance between selecting the dose range across different pediatric age groups and maintaining formulation flexibility is essential, and this can only be achieved through close communication and collaboration among multidisciplinary teams of clinical pharmacologists, formulation scientists, pharmacometricians, and clinicians to ensure that appropriate formulations and dose flexibility are built to enable successful clinical execution.

It is essential to recognize what dose scaling methodologies cannot achieve. Dose scaling does not replace clinical judgment in individual patient care, and exposure matching does not guarantee therapeutic efficacy or safety in all patients. For neonates and very young infants, drug-specific ontogeny and formulation effects often dominate PK behavior in ways that current models cannot fully capture. Substantial evidence gaps remain for rare pediatric diseases and biologics, where limited clinical data constrain model development and validation. Dose scaling approaches do provide a framework for informed decision-making but require ongoing refinement as new data emerge. Recommendations for preterm neonates rest on a limited primary evidence base, reflecting the small number of drug development programs that have specifically studied this population, and require drug- and pathway-specific validation before clinical application.

Several limitations of this review should be acknowledged. As a narrative rather than systematic review, the included literature reflects the authors’ selection rather than a pre-specified search strategy and may carry corresponding selection and citation bias. The practical guidance provided reflects the authors’ current interpretation of published evidence across the drug classes considered but should not be assumed to apply uniformly across all therapeutic areas, mechanisms of CL, or clinical settings. Finally, pediatric pharmacology and its regulatory framework are evolving rapidly; several of the recommendations summarized here are likely to require revision as new ontogeny data, modeling approaches, and regulatory guidance emerge.

## 8. Practical Recommendations

Based on the evidence reviewed, we propose decision rules for pediatric dose scaling, organized by scaling approach (Section 8.1), specific populations (Section 8.2), and regulatory submissions (Section 8.3). Each recommendation carries a qualitative evidence-strength tag: well supported denotes consistent evidence from multiple pediatric studies or established regulatory guidance; moderately supported denotes reasonable but more limited or indirect evidence; and expert opinion/limited evidence denotes recommendations resting largely on mechanistic reasoning or sparse data. A formal Grading of Recommendations Assessment, Development and Evaluation (GRADE) assessment, the internationally recognised system for rating the certainty of evidence and the strength of recommendations [124], was beyond the scope of this narrative review but would be a useful future direction. Recommendations specific to preterm neonates rest on a limited primary evidence base and are flagged accordingly.

### 8.1. Recommendations by Scaling Approach

We make the following recommendations:

1. Renally cleared drugs in children younger than 2 years: prefer allometric scaling with a maturation function, or PBPK modeling, over simple mg/kg dosing, because simple weight-based dosing does not capture renal maturation. (well supported)
2. Older children (>5 years) with normal body composition: simple mg/kg dosing may remain acceptable when adult data support linear PK–weight relationships; the higher age threshold reflects the fact that mg/kg dosing does not correct the nonlinear PK–weight relationship that allometric scaling captures, and is therefore reserved for the school-age range in which that nonlinearity is small. (well supported)
3. First-in-pediatric dose selection when mechanistic understanding is critical: use PBPK modeling, which integrates age-dependent physiology and ontogeny and is increasingly accepted by regulators. (moderately supported)

### 8.2. Recommendations for Specific Populations

The recommendations below are summarised in Supplementary Figure S1, which maps each special population to its recommended scaling approach, preferred sizing basis, and monitoring strategy using the same colour and certainty scheme as depicted in Figure 2.

1. Neonates and preterm infants: incorporate maturation functions (size plus PMA-based maturation) or preterm-specific PBPK models rather than allometry alone, and confirm predictions against emerging data where possible [125]. (expert opinion/limited evidence; preterm-neonate evidence is limited)
2. Obese children: consider fat-free mass or other body-composition metrics rather than total body weight for many drugs, including lipophilic agents and drugs such as propofol, gentamicin, vancomycin, and busulfan, where TBW-based dosing has been associated with altered exposure. (moderately supported)
3. Critically ill children (augmented renal clearance, therapeutic hypothermia, or extracorporeal support): anticipate large, time-varying changes in clearance and volume of distribution that often dominate developmental effects and use therapeutic drug monitoring with model-informed precision dosing rather than empirical weight-based dosing. (moderately supported)
4. Narrow-therapeutic-index drugs: prioritize exposure matching and plan for therapeutic drug monitoring from the outset. (well supported)

### 8.3. Recommendations for Regulatory Submissions

We make the following recommendations for regulatory submissions:

1. Align the extrapolation strategy with ICH E11A: use exposure matching alone where disease and response similarity are well supported, and add pediatric-specific PD, efficacy, or safety data where uncertainty is greater. (well supported)
2. Choose PBPK versus PopPK according to the available data: favour PBPK for first-in-pediatric and rare-disease settings where clinical data are sparse, and PopPK where pediatric PK data exist for model refinement and TDM. Both should be supported by appropriate verification and validation. (moderately supported)
3. Translate modeling output into simple bedside rules: express dose as weight-band or age-and-weight-band regimens with explicit guidance for renal or hepatic dysfunction. Indicate the use of TDM, rather than equations to be evaluated in real time. Identify well before submission how the model-informed dose will be communicated to prescribers and dispensers, including pediatric-specific dosing tables and dose-rounding rules [90]. (well supported)

## 9. Future Directions

Model-informed drug development will increasingly guide pediatric dose selection across drug development phases. Integration of in silico, in vitro, and in vivo data into detailed PBPK and PopPK models will enable more accurate predictions, particularly for neonates and infants.

Novel biomarkers beyond SCr, including cystatin C and emerging kidney injury markers, may improve renal function estimation in young children. Machine learning approaches are being explored to identify optimal covariate relationships and improve the predictive performance of PopPK models. Real-world data from EHRs offer opportunities for post-marketing dose-optimization studies that were previously impractical. Digital twins represent an emerging evolution of model-informed precision dosing, in which mechanistic pharmacometric models are combined with individual patient physiological and clinical data to form a virtual patient that is updated as new data accrue, allowing simulation of drug exposure, prediction of therapeutic response and toxicity, and adaptive dose optimization across the pediatric age spectrum [121,126]. Recent reviews describe an emerging paradigm in which machine learning and artificial intelligence methods are integrated with PBPK and PK/PD modeling to support parameter estimation, virtual population generation, and uncertainty quantification [127]. Critical assessments emphasize, however, that these approaches must be evaluated against established pharmacometric standards, with explicit attention to interpretability, training-set diversity, and prospective validation before clinical adoption [128]. Trial-design methodology is also evolving: an accuracy-for-dose-selection framework has been proposed as an alternative to traditional parameter-precision criteria for justifying pediatric PK study designs, with the aim of more directly aligning trial designs with the regulatory question of selecting an appropriate dose [129].

Integration of adaptive dosing approaches with therapeutic drug monitoring (TDM), Bayesian forecasting, and model-informed precision dosing tools will further refine individualized treatment in clinical practice. Emerging technologies, including artificial intelligence, digital twins, and learning healthcare systems, are likely to complement established pharmacometric approaches by enabling continuous refinement of dosing recommendations as new patient data become available.

Looking ahead, MIPD, already established for narrow-therapeutic-index agents, is expected to broaden across drug classes in pediatric practice as it is integrated with transporter and enzyme ontogeny data and emerging real-world data sources, although randomized trials demonstrating clinical benefit are still needed. Recent reviews illustrate how PBPK modeling can complement MIPD by supplying mechanistic predictions of site-of-infection PK/PD targets, drug–drug interactions, and exposure in special subpopulations such as preterm neonates, obese children, and those with renal impairment that population models alone may not adequately capture [121].

Important gaps remain. Mechanistic data are still sparse for preterm neonates, transporter and enzyme ontogeny, biologics and immunogenicity in young children, pediatric PD, and disease states such as ARC and therapeutic hypothermia. Continued progress will depend on generating high-quality pediatric clinical data and refining ontogeny and disease-state physiology in pediatric PBPK platforms, prospectively validating model-informed approaches in clinical studies, and demonstrating that improved exposure prediction translates into improved clinical outcomes across diverse pediatric populations.

Ultimately, the integration of therapeutic drug monitoring, Bayesian forecasting, model-informed precision dosing, artificial intelligence, digital twins, and learning healthcare systems has the potential to deliver increasingly individualized pediatric therapy. The goal is that every child receives a dose informed by the best available mechanistic, clinical, and regulatory evidence.

## 10. Conclusions

Pediatric dose selection has matured from a discipline reliant on empirical body-weight scaling to one in which allometry, maturation functions, PBPK modeling, and PopPK approaches are used in a complementary fashion, with the choice of method calibrated to age, available data, and CL pathway. The framework is no longer purely PK: developmental PD, age-appropriate formulation and excipient safety, ADA risk in pediatric biologics, drug–drug interactions, and TDM are now integral to the dose-selection conversation, as are the historical lessons regarding chloramphenicol, the sulfonamides, and codeine that motivated developmentally informed dose selection in the first place. These approaches should be viewed as complementary rather than competing methodologies, often applied sequentially and increasingly integrated across the drug development lifecycle.

The formalization of pediatric extrapolation under ICH E11A, the expanding regulatory acceptance of PBPK for neonatal and rare-disease indications, and the emergence of MIPD have together moved the field from generic dose recommendations toward population- and patient-specific exposure targets.

## Figures and Tables

**Table 1:** Comparison of Pediatric Dose Scaling Methods.

Method	Approach	Advantages	Limitations	Typical Use Cases	Regulatory/Dossier Use
Simple mg/kg [24,25]	Linear scaling by TBW	Simple; widely used; easy to calculate	Does not account for the nonlinear PK–weight relationship; inaccurate in obesity	Quick estimates; older children with normal body composition	Common in pediatric labels for older children, particularly for broad-therapeutic-index drugs with established linear PK; not generally relied upon as a primary basis for first-in-pediatric dose justification
BSA-based [6]	Dose per m^2^ BSA	Traditional for chemotherapy; historically rationalized by correlation with metabolic rate (rationale now considered less favored for new pediatric applications)	Not superior to allometry; calculation complexity; not validated <1 year	Oncology (historical); some biologics	Historically used in some pediatric oncology labels; generally discouraged for new pediatric labels in the absence of a specific mechanistic rationale
Allometric (0.75) [6,26]	CL = CL_std_ × (WT/70)^0.75^	Biologically motivated; accounts for the nonlinear metabolism–size relationship	Inadequate <2 years without maturation function; exponent debated	Children > 2 years; initial estimates across the age range	Widely used in dossiers for CL prediction and bridging in children beyond about 2 years; typically combined with a maturation function for younger ages
Allometric + Maturation [6,27]	CL = CL_std_ × F_size_ × F_mat_	Accounts for developmental changes; validated for renal and hepatic clearance maturation	Requires maturation parameters; drug-specific validation needed	Neonates and infants; renally cleared drugs	Standard approach for first-in-pediatric CL prediction in neonates and infants, especially for renally cleared drugs; well established in regulatory practice
FFM-based [28,29]	Scaling by FFM rather than TBW	Better correlation with CL in obesity; more physiological	Requires FFM estimation; pediatric extensions available (e.g., Al-Sallami equation)	Obese children; drugs whose distribution favors lean tissue	Increasingly applied within dossiers for obese children and as a covariate in PopPK and PBPK analyses, rather than as a stand-alone labeling basis
PBPK modeling [7,30]	Mechanistic model with age-specific physiology	Integrates multiple processes; can predict in untested populations using prior knowledge of physiology and drug properties; accepted by regulators	Complex; requires expertise; software costs; validation challenges	First-in-pediatric dose selection; regulatory submissions	Widely accepted by regulators for pediatric dose selection, DDI assessment, and labeling, including first-in-pediatric and rare-disease settings
PopPK modeling [8]	Data-driven model with covariates	Uses sparse data; quantifies variability; supports TDM	Requires pediatric PK data; extrapolation limited	Dose optimization with clinical data; TDM applications	Widely accepted for pediatric labeling and extrapolation across age groups; the most common analysis supporting neonatal and pediatric dosing in dossiers
Practical guidance: Simple mg/kg dosing is acceptable for older children with normal body composition when adult data support linear scaling. Allometric scaling combined with a maturation function is preferred for neonates and infants, especially for renally cleared drugs. FFM-based scaling is preferred for obese children. PBPK modeling is preferred for first-in-pediatric dose selection or when a mechanistic understanding of ontogeny is critical. PopPK modeling is preferred when pediatric PK data are available for model refinement and therapeutic drug monitoring. Here, “preferred” denotes the approach judged most suitable for a given age or pathway by weight of evidence and regulatory experience, rather than a formally graded ranking.					

Abbreviations: PK, pharmacokinetic; BSA, body surface area; CL, clearance; CLstd, standardized clearance; WT, weight; FFM, fat-free mass; TBW, total body weight; PBPK, physiologically based pharmacokinetic; PopPK, population pharmacokinetic; TDM, therapeutic drug monitoring; Fsize, size function; Fmat, maturation function.

**Table 2:** Serum Creatinine-Based GFR Estimation Equations for Pediatric Patients.

Equation	Formula	k Values	Notes/Applicability
**Pediatric-derived/validated equations (preferred in children and adolescents)**			
Original Schwartz [47]	eGFR = k × HT/SCr	k = 0.33 (LBW/preterm infants); k = 0.45 (term infants < 1 y); k = 0.55 (children 1–13 y, both sexes, and adolescent females ≥ 13 y); k = 0.70 (adolescent males ≥ 13 y)	Original equation. Overestimates GFR by approximately 20–25% with modern IDMS-traceable creatinine assays. HT in cm, SCr in mg/dL.
Bedside Schwartz [44]	eGFR = 0.413 × HT/SCr	k = 0.413 (all ages)	IDMS-traceable enzymatic creatinine. Most widely used for children 1–16 years; validated primarily in CKD populations; not validated for children < 1 year. HT in cm, SCr in mg/dL.
Counahan–Barratt [48]	eGFR = 0.43 × HT/SCr	k = 0.43 (all ages)	Pediatric alternative to Schwartz; single fixed k simplifies calculation. HT in cm, SCr in mg/dL.
Léger [49]	eGFR = (56.7 × WT + 0.142 × HT^2^)/SCr	N/A	Incorporates both weight and height and returns absolute GFR in mL/min (not BSA-normalized). Derived in children using Jaffé-method creatinine in µmol/L (not IDMS-traceable mg/dL); WT in kg, HT in cm.
CKiD Combined Creatinine–cystatin C [50]	eGFR = 39.8 × (HT/SCr)^0.456^ × (1.8/CysC)^0.418^ × (30/BUN)^0.079^ × 1.076^male^ × (HT/1.4)^0.179^	N/A	2012 immunonephelometric CKiD equation (distinct from the 2009 turbidimetric version, which uses different coefficients). Uses creatinine, cystatin C, and BUN; most accurate but requires multiple biomarkers. HT in metres for this equation (cm for all other equations in this table); SCr in mg/dL, CysC in mg/L, BUN in mg/dL; male = 1, female = 0.
**Adult-derived equations (not recommended for routine pediatric use; included for extrapolation context only)**			
Lund–Malmö Revised (LMR) [51,52]	eGFR = exp(X − 0.0158 × age + 0.438 × ln(age)), where the following definitions are used: Female, SCr < 150 µmol/L: X = 2.50 + 0.0121 × (150 − SCr) Female, SCr ≥ 150 µmol/L: X = 2.50 − 0.926 × ln(SCr/150) Male, SCr < 180 µmol/L: X = 2.56 + 0.00968 × (180 − SCr) Male, SCr ≥ 180 µmol/L: X = 2.56 − 0.926 × ln(SCr/180)	N/A (sex- and creatinine-band specific; see formula)	Adult-derived (Swedish cohort). SCr in µmol/L and age in years for this equation (not mg/dL). Not recommended for routine pediatric eGFR; applicable to children only after adjusting childhood creatinine to an age-18 equivalent [53]. Included only for adult-to-pediatric extrapolation context.
Cockcroft–Gault [54]	CLcr = ((140 − age) × WT)/(72 × SCr) × 0.85 if female	N/A	Estimates creatinine clearance (CLcr), not GFR. Adult-derived. FDA-recommended for renal-impairment dose adjustment but not physiologically optimal in adolescents; sensitive to body weight. Age in years, WT in kg, SCr in mg/dL.

Units: Unless otherwise noted in the table, HT is in cm and SCr in mg/dL. The Léger and Lund–Malmö equations were derived with creatinine in µmol/L, and the CKiD equation uses HT in metres; these per-equation units are stated in the Notes column. Pediatric applicability: Pediatric-derived equations (top group) should be preferred in children and adolescents. The adult-derived equations (bottom group) are not recommended for routine pediatric use and are included only for adult-to-pediatric extrapolation and FDA renal-impairment labeling in older adolescents and adults. Abbreviations: eGFR, estimated glomerular filtration rate (mL/min/1.73 m^2^); HT, height; SCr, serum creatinine; WT, body weight (kg); CLcr, creatinine clearance; LBW, low birth weight; IDMS, isotope dilution mass spectrometry; CysC, cystatin C; BUN, blood urea nitrogen; CKiD, Chronic Kidney Disease in Children; LMR, Lund–Malmö Revised; CKD, chronic kidney disease.

**Table 3:** Examples of PBPK and PopPK Modeling Applications in Pediatric Drug Development.

Drug	Approach	Application	Age Group	Outcome
Acetaminophen	PBPK	PK prediction in young children	Neonates, infants	Predictions within two-fold of observed concentrations across studied age groups [75]
Remdesivir	PBPK	Pediatric COVID-19 dose selection	Neonates to adolescents (≥28 days, ≥3 kg)	PBPK-derived weight-based regimen supported emergency use authorization, compassionate use, and the subsequent FDA pediatric approval (April 2022) [76]
Oseltamivir	PBPK	Prodrug PBPK model incorporating metabolic and renal maturation	Neonates, infants	Predicted oseltamivir and active-metabolite exposure in neonates and infants, consistent with observed data [77]
Sotalol	PBPK	Full age continuum modeling (sotalol as a model drug)	Neonates to adults	Adequate prediction across pediatric age groups, with reduced accuracy in neonates [78]
Dabigatran etexilate	PopPK + E-R	Pediatric VTE treatment; age- and weight-adjusted dosing nomogram evaluated in the DIVERSITY trial	Birth to <18 years	Population PK and exposure–response analyses supported the age- and weight-stratified dosing algorithm and FDA pediatric approval [79]
Adalimumab	PopPK	Adolescent HS approval	Adolescents (≥12 years, ≥30 kg)	Approval extended to adolescents based on PopPK simulations supporting exposure matching to adults [80]
Esomeprazole	PopPK + E-R	Pediatric GERD dosing	1 month to 17 years	Exposure-matching modeling supported approval across the pediatric age range [81]
Canakinumab	PopPK	Periodic fever syndromes	<2 years	Dosing extrapolated to the youngest patients [82]
Linezolid	PBPK	Neonatal PK prediction	Neonates, infants	Provided exposure predictions in age groups where PopPK data were sparse [83]

Abbreviations: PBPK, physiologically based pharmacokinetic; PopPK, population pharmacokinetic; PK, pharmacokinetic; E-R, exposure–response; VTE, venous thromboembolism; HS, hidradenitis suppurativa; GERD, gastroesophageal reflux disease; FDA, US Food and Drug Administration.

**Table 4:** Harmonized Comparison of Pediatric Age Classifications Used by ICH, FDA, and EMA.

Age Category	ICH E11/E11(R1) Definition (a)	FDA Interpretation (b)	EMA/EU Practice (c)
Preterm newborn infants	Born <37 weeks gestational age. For preterm newborns, the neonatal period extends from the day of birth through the expected date of delivery plus 27 days	Considered separately from term neonates in pediatric study plans; gestational age and postnatal age both required	Aligned with ICH E11(R1); preterm subgroups frequently stratified by gestational age in PIPs
Term newborn infants (neonates)	0 to 27 days postnatal age, ≥37 weeks gestational age	Aligned with ICH E11(R1); neonatal studies require dedicated rationale per FDA guidance	Aligned with ICH E11(R1)
Infants and toddlers	28 days to 23 months	Aligned with ICH E11(R1); some FDA documents subdivide at 12 months for formulation considerations	Aligned with ICH E11(R1)
Children	2 to 11 years	Aligned with ICH E11(R1)	EU practice commonly subdivides into preschool children (2 to 5 years) and school-age children (6 to 11 years) for clinical study design
Adolescents	12 to 16–18 years (dependent on region)	Birth to “younger than 17 years old” per 21 CFR 201.57(c)(9)(iv)(A); FDA pediatric population therefore caps at <17 years	EU and ICH define adolescents up to 18 years; EMA commonly applies an upper bound of 18 years

Compiled from ICH E11/E11(R1), FDA, and EMA guidance documents. (a) ICH E11 (December 2000) and ICH E11(R1) Addendum (Step 4, 18 August 2017; effective in the EU 28 February 2018; FDA finalization April 2018), age classification of pediatric patients (ICH, 2000, 2017) [97,100]. (b) FDA interprets the pediatric population per 21 CFR 201.57(c)(9)(iv)(A) (“birth to 16 years,” i.e., younger than 17 years old) and the Guidance for Industry: Pediatric Information Incorporated Into Human Prescription Drug and Biological Product Labeling (U.S. Food and Drug Administration, March 2019) [80,98]. (c) EMA reference EMA/CPMP/ICH/2711/1999; legal effective date 28 February 2018 (European Medicines Agency, 2018) [100]. EU subdivisions per the European Commission paediatric framework, Regulation (EC) No 1901/2006 (European Commission, 2006) [101]. Abbreviations: ICH, International Council for Harmonisation of Technical Requirements for Pharmaceuticals for Human Use; FDA, US Food and Drug Administration; EMA, European Medicines Agency; PIP, Paediatric Investigation Plan; CFR, Code of Federal Regulations [97,100].

## Data Availability

No new data were created or analyzed in this study. Data sharing is not applicable to this article.

## Supplementary Materials

The following supporting information can be downloaded at https://www.mdpi.com/article/10.3390/ph19071090/s1, Figure S1: Recommended dose scaling strategy by special pediatric population. Matrix mapping each special pediatric population (neonates and preterm infants; obese children; critically ill children with augmented renal clearance, therapeutic hypothermia, or extracorporeal support; and narrow-therapeutic-index drugs) to its recommended scaling approach, preferred sizing basis, and monitoring strategy. Cell colour indicates the strength of the recommendation for that approach in the stated population; the dot rating indicates the authors’ certainty in the supporting evidence, using the same colour and certainty scheme as Figure 2.

## Abbreviations

The following abbreviations are used in this manuscript:

**Abbreviation**	**Definition**
AAG	alpha-1-acid glycoprotein
ADA	anti-drug antibody
ADME	absorption, distribution, metabolism, and excretion
AI	artificial intelligence
ARC	augmented renal clearance
BCRP	breast cancer resistance protein
BMI	body mass index
BSA	body surface area
BUN	blood urea nitrogen
CFR	Code of Federal Regulations
CKD	chronic kidney disease
CKiD	Chronic Kidney Disease in Children (study)
CL	clearance
CLcr	creatinine clearance
CPIC	Clinical Pharmacogenetics Implementation Consortium
CYP	cytochrome P450
CysC	cystatin C
DDI	drug–drug interaction
ECMO	extracorporeal membrane oxygenation
eGFR	estimated glomerular filtration rate
EHR	electronic health record
EMA	European Medicines Agency
E-R	exposure–response
EU	European Union
FcRn	neonatal Fc receptor
FDA	U.S. Food and Drug Administration
FFM	fat-free mass
GA	gestational age
GERD	gastroesophageal reflux disease
GFR	glomerular filtration rate
GRADE	Grading of Recommendations Assessment, Development and Evaluation
HS	hidradenitis suppurativa
HT	height
ICH	International Council for Harmonisation of Technical Requirements for Pharmaceuticals for Human Use
IDMS	isotope dilution mass spectrometry
IQ	International Consortium for Innovation and Quality in Pharmaceutical Development
K/DOQI	Kidney Disease Outcomes Quality Initiative
LBW	low birth weight
mAb	monoclonal antibody
MIDD	model-informed drug development
MIPD	model-informed precision dosing
mRNA	messenger RNA
NPDE	normalized prediction distribution error
NUDT15	*nudix hydrolase 15*
OATP	organic anion transporting polypeptide
PBPK	physiologically based pharmacokinetic
PD	pharmacodynamic
PGx	pharmacogenetic
PIP	Paediatric Investigation Plan
PK	pharmacokinetic
PK/PD	pharmacokinetic/pharmacodynamic
PMA	postmenstrual age
PopPK	population pharmacokinetic
SCr	serum creatinine
SD	standard deviation
STEP	Safety and Toxicity of Excipients for Paediatrics (database)
TBW	total body weight
TDM	therapeutic drug monitoring
TPMT	*thiopurine S-methyltransferase*
UDP	uridine diphosphate
UGT	UDP-glucuronosyltransferase
Vd	volume of distribution
VPC	visual predictive check
VTE	venous thromboembolism
WT	body weight
