# Supplementary figures and images for: Advancing Pediatric Dose Scaling: Strategies, Modeling Approaches, and Clinical Applications
Source: Pharmaceuticals (Basel). 2026 Jul 15;19(7):1090. doi: 10.3390/ph19071090 (PMC13415250; doi:10.3390/ph19071090)

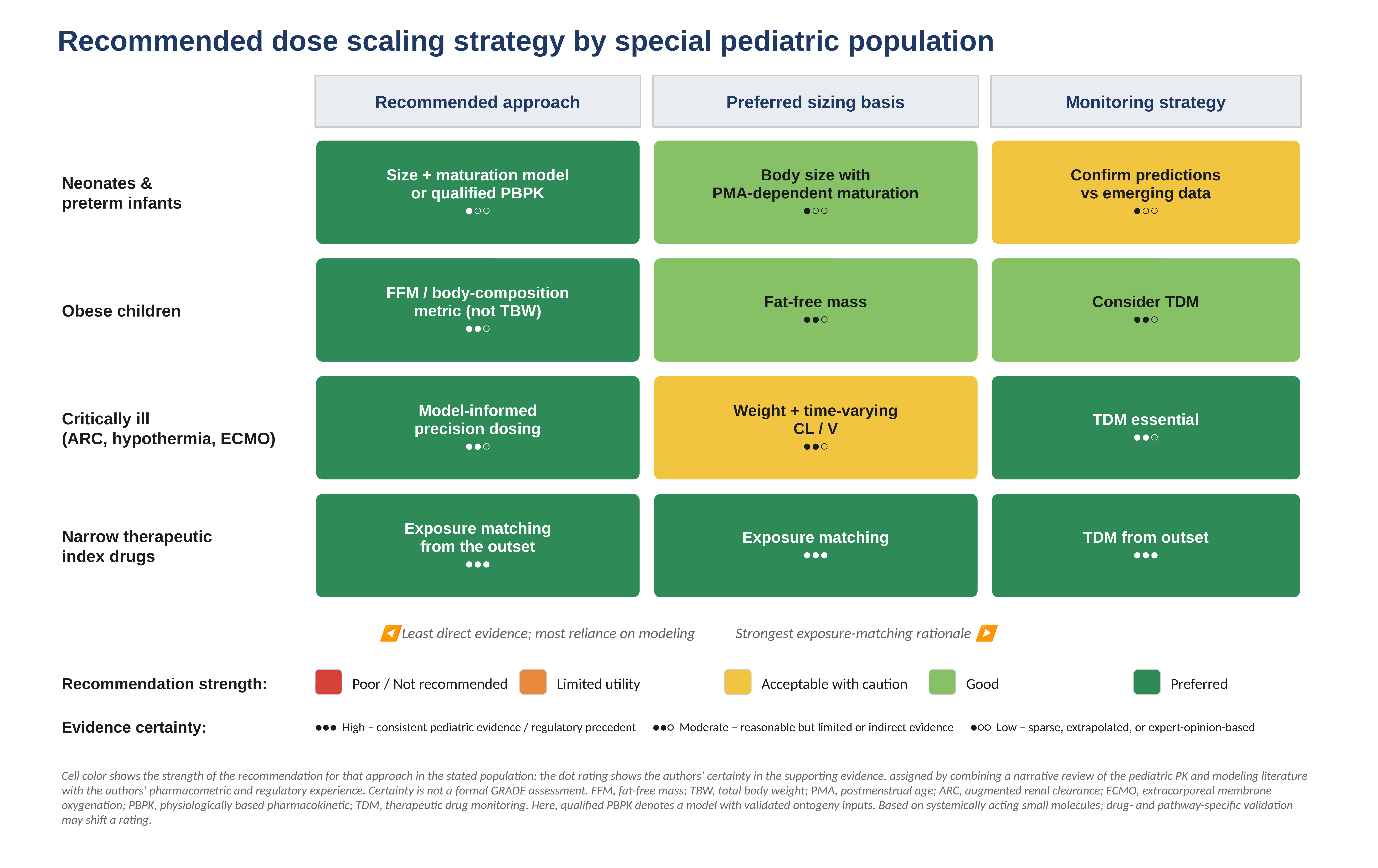

Supplement: Supplementary file 1 [file pharmaceuticals-19-01090-s001.zip › pharmaceuticals-4376605-supplementary.png]
